# Supplementary material for: Patterns of Use of Vaping Products among Smokers: Findings from the 2016–2018 International Tobacco Control (ITC) New Zealand Surveys
Source: Int J Environ Res Public Health. 2020 Sep 11;17(18):6629. doi: 10.3390/ijerph17186629 (PMC7559110; doi:10.3390/ijerph17186629)
Supplement: Supplementary file 1 [file ijerph-17-06629-s001.pdf]

Table S1. E-cigarette related questions in the Wave 2 of the ITC NZ Survey.

Question	Response options	Notes
Awareness, trial and use of e-cigarettes		
Have you ever heard of electronic cigarettes, also called e-cigarettes, vapes, or vaping devices?	Y/N	
Have you ever used an e-cigarette or vaping device, even one time?	Y/N	Asked of participants who were aware of e-cigarettes
On average, how often do you currently use e-cigarettes or vaping devices?	•Every day<br>•At least once a week, but not every day<br>•At least once a month, but not every week<br>•Less than monthly<br>•Not at all	Asked of participants who had used e-cigarettes in the last year
Type of products used and place of purchase		
Which of the following best describes the type of e-cigarette or vaping device you (weekly or daily users)/ used last (monthly users)?		Asked if currently use e-cigarettes at least monthly
Does the e-cigarette or e-liquid that you currently use most contain nicotine? (weekly or daily users)<br>Did the e-cigarette or e-liquid that you used last contain nicotine? (monthly users)	Y/N<br>Y/N	Asked if currently use e-cigarettes at least monthly
What flavours of e-cigarette or e-liquid have you used in the last 30 days?	•Unflavoured e-liquid<br>•Tobacco flavour<br>•Mix of tobacco and menthol<br>•Menthol or mint<br>•Fruit flavour<br>•Candy, desserts, sweets<br>•Chocolate<br>•Other	Asked if currently use e-cigarettes at least monthly
Of the flavours you use, which do you use most often?	•Unflavoured e-liquid<br>•Tobacco flavour<br>•Mix of tobacco and menthol<br>•Menthol or mint<br>•Fruit flavour<br>•Candy, desserts, sweets<br>•Chocolate<br>•Other	Asked if currently use e-cigarettes at least monthly
Now thinking about the last time you purchased e-liquid, e-cigarette cartridges or disposable e-cigarettes. Where did you make this last purchase?	•On the internet<br>•Vape shop/e-cigarette kiosk<br>•Tobacco specialty shop/ tobacconist	Asked if currently use e-cigarettes at least monthly

	•Newsagent/ bottle-store/ dairy or convenience store<br>•(Petrol/ Gas) station shop<br>•Supermarket<br>•Chemist/ Pharmacy<br>•Pub or Bar<br>•Other store<br>•Independent seller, not a mainstream shop, market stall, pop-up shop<br>•Bought abroad and brought back with me<br>•Friend or relative<br>•Free sample<br>•Shared bulk purchase with other people<br>•Somewhere else	
Reasons and motivators for using vaping product		
Which of the following are, or were, reasons for your use of e-cigarettes or vaping devices?		Asked if currently use e-cigarettes at least monthly
Replacing some of your ordinary cigarettes with an e-cigarette or vaping device means you don't have to give up smoking ordinary cigarettes altogether	Y/N	
To make it easier to cut down on the number of ordinary cigarettes you smoke	Y/N	
As a way to help you quit smoking	Y/N	
They may not be as bad for your health	Y/N	
Because you can use them in places where smoking ordinary cigarettes is banned.	Y/N	
Using e-cigarettes or vaping devices is less harmful than smoking to other people around you	Y/N	
Using e-cigarettes or vaping devices is more acceptable than smoking to other people around you	Y/N	
You enjoy using e-cigarettes or vaping devices	Y/N	
You save money by using e-cigarettes or vaping devices instead of smoking	Y/N	
Attitudes and beliefs about vaping products		
Now I'm going to read out a list of statements about using e-cigarettes or vaping devices. Please tell me whether you agree or disagree with each statement		Asked if aware of e-cigarettes
E-cigarettes or vaping devices are too hard to get	•Strongly agree<br>•Agree<br>•Neither agree or disagree<br>•Disagree<br>•Strongly disagree	
E-cigarettes are complicated to use	•Strongly agree<br>•Agree	

	•Neither agree or disagree<br>•Disagree<br>•Strongly disagree	
Compared to smoking ordinary cigarettes, do you think using e-cigarettes or e-liquids that contain nicotine is…	•Much less addictive than smoking cigarettes<br>•Somewhat less addictive than smoking cigarettes<br>•Equally addictive to smoking cigarettes<br>Somewhat more addictive than smoking cigarettes<br>Much more addictive than smoking cigarettes	Asked if aware of e-cigarettes
Overall, as compared with smoking cigarettes, is (ever tried e-cigarettes) / do you think (never tried e-cigarettes) using e-cigarettes or vaping devices is…	•A lot cheaper<br>A bit cheaper<br>•About the same cost<br>•A bit more expensive<br>•Alot more expensive	Asked if aware of e-cigarettes
How satisfying is vaping (using e-cigarettes), compared to smoking ordinary cigarettes?	•Much less satisfying than smoking<br>Somewhat less satisfying<br>•Equally satisfying to smoking<br>•Somewhat more satisfying<br>•Much more satisfying than smoking	Asked if ever tried e-cigarettes
In your opinion, is using e-cigarettes or vaping devices less harmful to health, more harmful to health, or no different than smoking ordinary cigarettes?	•Much less harmful<br>•Somewhat less harmful<br>•No different<br>•Somewhat more harmful<br>•Much more harmful	Asked if aware of e-cigarettes

Table S2. Changes (absolute difference, 95% CI) in awareness and use of vaping products from Wave 1 to Wave 2 (supplement to Table 2 in paper).

Question	All Participants (W1 *n* = 1086; W2 *n* = 1020)		Smokers (W1 *n* = 857; W2 *n* = 726)		Ex-smokers (W1 *n* = 229; W2 *n* = 294)	
	Absolute Difference [1] (95% CI)	*p*-Value	Absolute Difference [1] (95% CI)	*p*-Value	Absolute Difference [1] (95% CI)	*p*-Value
Awareness of vaping products [2]	4.2 (2.2–6.2)	<0.001	4.2 (1.8–6.5)	<0.001	4.2 (0.8–7.5)	0.014
Ever used vaping products [2]	17.2 (12.7–21.8)	<0.001	17.5 (12.2–22.7)	<0.001	15.7 (6.3–25.1)	0.001
At least monthly current use of vaping products [3]	6.0 (1.5–10.6)	0.009	5.4 (0.2–10.5)	0.040	7.7 (−2.0–17.3)	0.119
Daily current use of vaping products [3]	3.1 (−0.2–6.4)	0.069	3.0 (−0.3–6.4)	0.078	2.9 (−6.1–12.0)	0.525

Note: W1—Wave 1; W2—Wave 2; CI—confidence interval; [1] Absolute differences are expressed as Wave 2 minus Wave 1 (i.e. positive values indicate increase). Estimates are from marginally standardised model, with standardisation across waves for age group, gender, ethnicity, and smoking status (smoking status adjustment for "All participants" row only). As the standardisation for changes is across both waves, the absolute differences here are slightly different from the absolute differences that can be calculated from Table 2 (which only include adjustment within wave); [2] Valid answers at Wave 1: *n* = 1086; Wave 2: *n* = 1020; [3] Valid answers at Wave 1: *n* = 1039; Wave 2: *n* = 1012.
